# Differing Contributions of Classical Risk Factors to Type 2 Diabetes in Multi-Ethnic Malaysian Populations

**DOI:** 10.3390/ijerph15122813

**Published:** 2018-12-10

**Authors:** Noraidatulakma Abdullah, Nor Azian Abdul Murad, John Attia, Christopher Oldmeadow, Mohd Arman Kamaruddin, Nazihah Abd Jalal, Norliza Ismail, Rahman Jamal, Rodney J. Scott, Elizabeth G. Holliday

**Affiliations:** 1 School of Biomedical Sciences and Pharmacy, Faculty of Health and Medicine, University of Newcastle, Newcastle 2308, New South Wales, Australia; noraidatulakma.abdullah@ppukm.ukm.edu.my (N.A.); rodney.scott@newcastle.edu.au (R.J.S.); 2 UKM Medical Molecular Biology Institute (UMBI), Universiti Kebangsaan Malaysia, Kuala Lumpur 56000, Malaysia; nor_azian@ppukm.ukm.edu.my (N.A.A.M.); arman@ppukm.ukm.edu.my (M.A.K.); jiha_jalal@yahoo.com (N.A.J.); norliza.ismail@ukm.edu.my (N.I.); 3 Clinical Research Design, IT and Statistical Support (CReDITSS) Unit, Hunter Medical Research Institute, Newcastle 2305, New South Wales, Australia; john.attia@newcastle.edu.au (J.A.); Christopher.Oldmeadow@hmri.com.au (C.O.); 4 Centre for Clinical Epidemiology and Biostatistics, School of Medicine and Public Health, Faculty of Health and Medicine, University of Newcastle, Newcastle 2308, New South Wales, Australia; 5 Hunter Area Pathology Service, John Hunter Hospital, Newcastle 2305, New South Wales, Australia

**Keywords:** type 2 diabetes, Asian populations, risk factor, genetic, rural, Malaysia

## Abstract

The prevalence of type 2 diabetes is escalating rapidly in Asian countries, with the rapid increase likely attributable to a combination of genetic and lifestyle factors. Recent research suggests that common genetic risk variants contribute minimally to the rapidly rising prevalence. Rather, recent changes in dietary patterns and physical activity may be more important. This nested case-control study assessed the association and predictive utility of type 2 diabetes lifestyle risk factors in participants from Malaysia, an understudied Asian population with comparatively high disease prevalence. The study sample comprised 4077 participants from The Malaysian Cohort project and included sub-samples from the three major ancestral groups: Malay (n = 1323), Chinese (n = 1344) and Indian (n = 1410). Association of lifestyle factors with type 2 diabetes was assessed within and across ancestral groups using logistic regression. Predictive utility was quantified and compared between groups using the Area Under the Receiver-Operating Characteristic Curve (AUC). In predictive models including age, gender, waist-to-hip ratio, physical activity, location, family history of diabetes and average sleep duration, the AUC ranged from 0.76 to 0.85 across groups and was significantly higher in Chinese than Malays or Indians, likely reflecting anthropometric differences. This study suggests that obesity, advancing age, a family history of diabetes and living in a rural area are important drivers of the escalating prevalence of type 2 diabetes in Malaysia.

## 1. Introduction

There are 425 million people living with diabetes worldwide and about 90 percent of these have type 2 diabetes (T2D) [1]. The prevalence of T2D is growing rapidly, especially in Asian countries, with about 60% of diabetes patients worldwide currently living in Asia [2].

Asians appear to be at higher risk of developing T2D than people of European ancestry [3]. While the causes are not entirely clear, recent demographic and lifestyle transitions are likely important. T2D prevalence in Asia has increased in parallel with rapid economic development, an ageing population, urbanisation, nutritional transitions and reduced physical activity [4]. The adoption of Westernised dietary patterns in Asian countries has been associated with increased T2D prevalence [5] and partly reflects greater caloric intake from animal fat, which has almost doubled in India and China in recent decades [6,7]. Asian populations also have the highest correlation between per capita sugar consumption and T2D prevalence among 165 countries, potentially suggesting greater sensitivity to glucose [8]. Compounding dietary changes, physical activity has also significantly decreased in Asian populations alongside rapid urbanization and modernisation [9,10].

In contrast to lifestyle changes, the contribution of known genetic variants to type 2 diabetes risk appears to be small in Asian populations. Recent genetic studies conducted in participants of East Asian ancestry suggests that known T2D genetic risk variants explain only about 2% of total variation in disease risk [11,12].

The estimated prevalence of diabetes in Malaysia has increased from 11.6% in 2006 to 17.5% in 2015 and it is estimated that over 3.5 million adults in Malaysia are living with diabetes [13]. Among Asian countries, Malaysia has one of the highest comparative prevalences of T2D in the Western-Pacific region, compared with other neighbouring countries such as Singapore (12.8%), Indonesia (6.2%), Philippines (6.1%) and Thailand (8.0%) [14]. In spite of this, this country has been relatively understudied in T2D research. Malaysia has a total population of 28.3 million [15] and its multi-ethnic society includes three major ancestral groups: Malay (~63%); Chinese (~25%); and Indian (~7%). The prevalence of T2D appears to differ among these groups, with Malaysian Indians having the highest prevalence (25 to 28%), followed by Malays (17 to 19%) and Chinese (9 to 14%) [16].

Similar to other Asian countries, the rising T2D prevalence in Malaysia appears unlikely to be the effect of known, common genetic risk variants. In a recent Malaysian study, a genetic risk score aggregating 62 validated T2D genetic risk variants explained less than 2% of overall T2D risk in any of the three major ancestral groups [17]. As in other Asian countries, lifestyle factors may be more important and quantifying their effect on population disease risk may help to identify targets for public health intervention. With this intent, the current study assessed the association and predictive utility of established T2D risk factors in case-control samples from the three major Malaysian ancestral groups. This study had two principal aims: (i) to assess the association between lifestyle risk factors and T2D, both within and across Malay, Chinese and Indian Malaysian populations; and(ii) to estimate and compare the combined contribution of all disease-associated risk factors to T2D within and between the three ancestral groups.

## 2. Materials and Methods

### 2.1. Data Sources and Study Samples

This was a nested case-control study using participants from the Malaysian Cohort project (TMC), a prospective multi-ethnic, population-based cohort including 106,527 volunteers aged between 35 and 70 years [16]. The original cohort sampling was performed using a mixed approach of voluntary participation together with cluster and targeted sampling. Participants were recruited from both rural and urban areas throughout Malaysia in April 2006 until the end of September 2012 [16]. Rural areas were chosen from settlements created by the government’s Federal Land Development Authority (FELDA) agricultural scheme, which was founded in 1956 with an initial focus on rubber and oil palm farming [16]. The FELDA cohort is a relatively non-mobile population, facilitating participant follow-up. A total 75 of 103 FELDA settlements were sampled throughout Malaysia and 25,907 invitations were sent to participants fulfilling eligibility criteria. A total 19,467 (75.1%) people responded and were recruited [16]. Participants from urban areas were recruited from publicity events held in cities, towns, government offices, private agencies and housing areas, as well as newspaper advertisements.

The current study was a nested case-control study of samples previously selected for large-scale genetic analysis, as described elsewhere [17]. Cases and controls were randomly selected from each major ancestral group (Malay, Chinese and Indian). Cases were defined based on fasting plasma glucose (FPG) exceeding 7.5 mmol/L (or 126 mg/dL), with a similar number of ancestry-matched controls defined based on FPG <5.5 mmol/L (or 99 mg/dL) and the absence of a previous diagnosis of diabetes. A slightly higher threshold of >7.5 mmol/L was used to define cases, to ensure cases were truly diabetic and reduce bias resulting from misclassification. Ethnicity was defined using the self- reported ethnicity of the participant and their family for three preceding generations.

A total of 4077 samples were used: 1323 Malays (600 cases, 723 controls), 1344 Chinese (654 cases, 690 controls) and 1410 Indians (708 cases, 702 controls). All participants provided written, informed consent for inclusion before they participated in the study. The study was conducted in accordance with the Declaration of Helsinki, and the protocol was approved by the Ethics Committee of Universiti Kebangsaan Malaysia (Project Code: FF-205-2007).

### 2.2. Risk Factor Selection

Known T2D risk factors were selected using evidence from previous studies [18,19]. These comprised age, gender, physical activity, sleep duration, body mass index (BMI), waist circumference (WC), waist-to-hip ratio (WHR), family history of diabetes (FHD) and location of study.

### 2.3. Questionnaire-Derived Variables

Information related to demographic and environment factors was collected at baseline using questionnaires and interviews [16]. FHD was determined by asking the participant whether they had a biological parent or sibling diagnosed with T2D and was categorised as “Yes” or “No”. Recruitment location was defined as rural or urban for each participant. Physical activity was classified based on the average, self-reported weekly vigorous activity [20] for the last four months (see Supplement for list of activities) and was categorised as “Active” or “Inactive” using a threshold of 150 min per week, as recommended by the World Health Organisation (WHO) [21]. Vigorous activity was used to avoid misclassification and bias. Sleep duration was defined based on self-reported values for weekdays (Monday to Friday) and weekends (Saturday and Sunday), with average daily sleep duration obtained as a weighted average: [(5xweekday duration) + (2xweekend day duration)]/7 [22]. As commonly performed in sleep research [23], sleep duration was classified into six categories (less than 6 h, 6 to 7 h, 7 to 8 h, 8 to 9 h, 9 to 10 h and more than 10 h) with 7 to 8 h chosen as the reference category [24]. Physical activity and sleep duration were assessed using the validated Malay International Physical Activity Questionnaire (IPAQ-M) [25].

### 2.4. Anthropometric Measurements

Height, weight, waist circumference (WC) and hip circumference were each measured three times and averaged. Body mass index (BMI) and waist-to-hip ratio (WHR) were calculated using the average values of height, weight, WC and hip circumference. BMI was categorised as: <25 kg/m^2^ (normal), 25–30 kg/m^2^ (pre-obese) and >30 kg/m^2^ (obese) [26]. A small number of underweight participants (BMI <18.5 kg/m^2^; n = 84) were combined with those of normal BMI, for reasons of parsimony. WHO cut-offs for BMI were used to permit comparisons with international studies of both Asian and non-Asian populations. For WC and WHR, sex-specific cut-offs recommended by the WHO were used to derive three categories [27]. For WC these were: low risk (males: <94 cm; females: <80 cm); moderate risk (males: 94–102 cm; females: 80–88 cm); and high risk (males: >102 cm; females: >88 cm) [28].WHR was categorised as: low risk (males: <0.95; females: <0.80); moderate risk (males: 0.96–1.0; females: 0.81–0.85); and high risk (males: >1; females: >0.85) [29].

### 2.5. Missing Data Handling

Both complete case and multiple imputation analyses were performed. Multiple imputation was performed by chained equations (MICE) with 25 cycles, based on a missing at random (MAR) assumption [30]. In each cycle, missing values for each variable were imputed based on a predictive distribution derived from regression on all other variables in the imputation model (gender, age group, WHR, FHD, location of study, sleep duration and physical activity). Parameter estimates from the imputed datasets were combined using Rubin’s rules [31].

### 2.6. Statistical Analyses

Multivariable logistic regression modelling was used to investigate associations between putative risk factors and T2D within each of the three ancestral groups, and in the combined group, adjusting for ethnicity. For each analysis a variable selection process was used as previously described [32]. This involved initially fitting a multivariable model including all selected risk factors, and then individually removing the least significant risk factor (*p* > 0.20) provided the likelihood ratio *p*-value for the nested models exceeded 0.20 and the estimated coefficients (on the logit scale) of all remaining variables did not differ by more than about 10%. To ensure final models were comparable across ancestral groups, any risk factor that had been removed for that ancestral group but retained for any other group was re-included in the final model. In primary analyses, WHR was treated as a continuous variable. Secondary analyses assessed models including the categorical WHR; these models did not adjust for gender to prevent adjusting twice for this variable, since WHR was classified using gender-specific cut-points. Models for the combined sample were adjusted for ethnicity. Secondary analyses also assessed multiplicative interaction between risk factors and ancestral group. Interaction analyses were performed only in complete-case data.

Parameter estimates were expressed as odds ratios (OR) with 95% confidence interval (CI). A threshold of 0.05 was used for declaring significance. The risk explained by the classical risk factors was estimated using McFadden’s pseudo R^2^. Based on the final model for each ancestral group, the Area Under the Receiver-Operating Characteristic curve (AUC) was calculated with its 95% confidence interval.

Based on the available sample sizes, the sample for each ancestral group offered approximately 80% power at α = 0.05 to detect an odds ratio of 1.36 for a risk factor with 0.35 prevalence in controls. For risk factors with prevalence of 0.25 or 0.15, each sample had approximately 80% power to detect odds ratios of 1.40 and 1.48, respectively. All analyses were performed using STATA 13.1 (Stata Corporation, College Station, TX, USA).

## 3. Results

Baseline characteristics for the full sample (n = 4077) are shown in Table 1. The corresponding statistics for participants used in complete-case analyses (n = 2247) are shown in Supplementary Table S1. Missing data was substantively due to the physical activity and sleep duration variables, resulting from a transition between two versions of physical activity questionnaires (which also measured sleep) during the study. In this sample, there was a difference in the gender distribution of cases and controls in all ancestral groups, with male participants being more likely to be a case than a control. Those aged 50–60 years were also more likely to be a case, compared to participants aged less than 50 or above 60 years. In contrast, participants aged under 50 were more likely to be a control. The sample included a preponderance of participants from urban areas. In Malay and Indian participants, participants from rural areas were more likely to be a case than a control, while those from urban areas were more likely to be a control.

A family history of diabetes (FHD) was associated with case-control status in Chinese and Indians; participants with a family history were more likely to be cases. BMI was associated with case-control status in Malay and Chinese, with overweight (BMI 25–29.9 kg/m^2^) and obese (BMI >30 kg/m^2^) participants being more likely to be a case. WHR showed strong association with case-control status in all three groups: participants with low risk WHR were more likely to be controls, and those with high risk WHR were more likely to be cases. Physical inactivity showed inconsistent patterns across ancestral groups, with no clear associations apparent. Similarly, sleep duration showed inconsistent trends; some association was observed in the Indian group with controls being more likely to have short sleep duration, and cases being more likely to have long sleep duration.

The final multivariable model included gender, age, waist-to-hip ratio, FHD, location of study, sleep duration and physical activity (Table 2). WC was removed from the model due to high collinearity with WHR, and because WHR has been established as a superior predictor of diabetes risk [33,34,35]. BMI did not explain significant variation in the outcome when WHR was included in the model so was also removed. None of the other risk factors showed association with T2D (at *p* < 0.20) or were retained in the multivariable model.

In complete case analyses, gender was not significantly associated with disease in any ancestral group after adjusting for other risk factors (Table 2). Older people had significantly higher odds of T2D than younger people in all ancestral groups. Compared to participants aged <50, those aged 50 to 60 years had odds ratios of 2.48 in Malay (95%CI: 1.77, 3.48), 2.62 in Indian (95%CI: 1.87, 3.68) and 1.11 in Chinese (95%CI: 0.70, 1.78). For participants aged >60, the highest odds ratio was observed in Chinese followed by Indian and Malay, although all confidence intervals overlapped. Higher WHR ratio was significantly associated with the odds of T2D in all groups. Physical inactivity was not significantly associated with disease in any group, although point estimates of odds ratios were mostly >1. Those who lived in rural areas had higher odds of T2D in all groups, with the association being significant in Malay and Indian. A family history of diabetes was associated with T2D in Chinese and Indian but not in Malay. Longer sleep duration (more than 10 h) was associated with T2D in Indian participants (OR: 7.10, 95% CI: 2.38, 21.17).

In the analysis of all groups combined, results were similar, with T2D odds being significantly higher in those of greater age, higher WHR, located in a rural area and with a positive family history. Results from multiply imputed data (n = 4077) were broadly similar to those of the complete case analysis but showed smaller standard errors and tighter confidence intervals (Table 3). The alternative model based on a categorical WHR for both complete case and multiply imputed analyses yielded analogous results, (Supplementary Tables S2 and S3).

### History of Diabetes

Based on the pseudo-R^2^, the combination of gender, age, WHR and physical inactivity, location, positive FHD and average sleep duration explained about 16% of variation in case-control status in Malay, 29% in Chinese and 22% in Indian (Table 2). In the combined sample, these seven factors explained an estimated 21% of case-control variation. Results from multiply imputed data were similar (Table 3).

**Figure 1:**
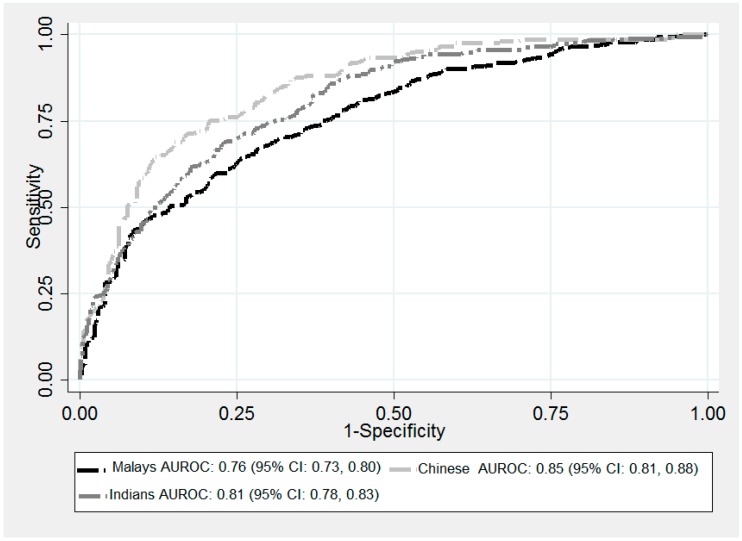
Receiver operating characteristic (ROC) curves with 95% CI for each Malaysian ancestral group based on complete-case analyses (n = 2247). AUC for combined group: 0.80 (0.78, 0.82).

The area under the receiver operating characteristic curve (AUC) was highest in Chinese (AUC: 0.85, 95% CI: 0.81, 0.88), followed by Indian (AUC: 0.81, 95% CI: 0.78, 0.83) and Malay (AUC: 0.76, 95% CI: 0.73, 0.80) (Figure 1). In the combined group, the AUC was 0.80 (0.78, 0.82). A test of equality of AUC estimates for the individual ancestral groups [36] showed a globally significant difference (*p* = 0.0018). In pairwise comparisons, the Chinese AUC was significantly different to the Malay (*p* = 0.0004). However, there was little difference between AUC estimates in Malay and Indian (*p* = 0.052) or Chinese and Indian (*p* = 0.065) (Table 4).

Tests of interaction between ancestral group and risk factors are shown in Table 5. Significant (*p* = 0.009) multiplicative interaction was observed between ancestry and intermediate age (50–60, compared to <50) where the effect in Chinese was significantly lower than in Malays (OR: 0.52 95% CI: 0.32, 0.85). Significant interaction was also observed between ancestry and gender, with the effect of being female in both Chinese and Indians being lower than the effect in Malays. Alternatively, there was significant interaction between waist-to-hip ratio and ancestry, with the effect of high-risk waist-to-hip ratio being greater in Chinese and Indians than the reference group of Malays (Chinese OR = 2.10, *p* = 0.006; Indians OR = 1.24, *p* = 0.36), although the result was not significant in Indians. The effect of being physically inactive in Indians was significantly lower than Malays but non-significantly lower in Chinese than Malays. There was no evidence of interaction between ancestral group and locality. Having positive family history of T2D showed significant interaction effect being higher in both Indians and Chinese respectively than Malays (OR Indians: 2.43, OR Chinese: 2.96). Sleep duration between 6 to 7 h indicated significantly lower effect in Chinese than Malays (OR: 0.52). Conversely, sleep duration more than 10 h was associated with significantly higher risk in Indians than Malays (Table 5).

## 4. Discussion

This study showed that a combination of seven risk factors accounted for about 21% of case-control variation in T2D in this Malaysian sample: age, gender, elevated waist-to-hip ratio, physical inactivity, rural location, family history of diabetes and sleep duration. Waist-to-hip ratio and age showed consistent, significant associations with disease across ancestral groups. This suggests that major contributors to the increasing T2D prevalence in Malaysia are determinants of central obesity such as urbanisation, diet and physical inactivity, together with the ageing population, with additional potential contributions by family history and environmental influences such as rural residence.

Neither age nor family history is a modifiable risk factor. Other studies have also observed increased T2D incidence with advancing age [37]. This is at least partly due to age-related reductions in skeletal muscle mass (sarcopenia) and activation of glycogen synthase, and surges in visceral adiposity, leading to insulin resistance and glucose intolerance [38,39]. This study also showed that a positive family history increased the odds of T2D. This is consistent with T2D being heritable, meaning a component of disease risk is contributed by genetic variation transmitted through families. An earlier study conducted in Chennai, India also reported that a positive family history was associated with a three-fold greater risk of T2D [40]. A study among Chinese with normal glucose tolerance also showed that participants with a family history of diabetes had poorer insulin sensitivity and greater insulin resistance than those without a family history [41].

Abdominal obesity is an established risk factor for T2D. It can lead to insulin resistance which increases T2D risk, by increasing the secretion of non-esterified fatty acids and adipocytokines such as tumour necrosis factor-α, and reducing adiponectin [42,43,44,45]. Asian individuals have been reported to have a higher distribution of body fat around central organs in the abdominal area with concomitantly lesser muscle mass, compared to Europeans with the same healthy BMI or waist circumference [46]. Within Asian groups, previous studies found that body fat percentage tends to be naturally higher in Indians, followed by Malay and Chinese [47,48]. In the current sample, Chinese controls also had less adiposity than Malay or Indian, corresponding with the lower overall T2D prevalence observed in Chinese Malay. However, the odds of T2D resulting from increasing adiposity was greater in Chinese compared to the other ancestral groups. This is also supported by the significant, positive interaction between higher waist-to-hip ratio and Chinese ancestry. An analogous result was observed in Chinese participants in the Multi-Ethnic Study of Atherosclerosis [49]. These findings have public health significance, suggesting a greater risk of diabetes resulting from obesity in Chinese individuals. The discoveries may also reflect anthropometric differences between ancestral groups, suggesting the possible utility of ethnicity-specific anthropometric cut- points for estimating diabetes risk.

Rapid urbanisation accompanied by diet and lifestyle changes occurring across Asia and leading to obesity could be a pivotal factor increasing T2D prevalence [4,50]. However, the current study found that those living in rural areas had higher odds of T2D than those in urban areas. This is consistent with the higher prevalence of obesity among those living in rural compared to urban areas in this current sample (Table S4); a National Health Morbidity Survey in Malaysia also found the prevalence of T2D was higher in rural than urban areas [51]. Another study in Malaysia also found that residents in rural areas generally have a higher burden of cardiovascular risk factors compared to their urban counterparts [52]. There are also likely to be additional unmeasured factors contributing to urban/rural differences in risk. Similar rural/urban differences were found in a large US study, which reported higher prevalence of diabetes among respondents from rural, compared to urban, areas [51]. Correspondingly higher prevalences of diabetes risk factors such as poverty, obesity and tobacco use were also found in the rural areas, and may partly explain the findings [53]. It is possible that similar behavioural and socio-demographic differences underlie the results from this study.

Longer sleep duration, potentially a marker of a sedentary lifestyle, also showed association with T2D in this study. A previous meta-analysis of eleven prospective studies reported that for every 1-h increase in sleep duration above an optimal seven hours, the risk of developing T2D increased by 14% [24]. This was also supported by the Whitehall II study finding that every 2 h increment in daily sleep duration above 7 h sleep increased T2D risk by 65% [54]. Prolonged sleep duration may have a metabolic effect on proinflammatory cytokine production, inducing insulin resistance [55] and lowering glucose tolerance [56]. Long sleep duration is also associated with other T2D risk factors such as low socioeconomic status, depression and physically inactivity [24]. It is thus possible that the apparent effect of long sleep duration is partly attributable to other causal factors not measured in this study. This study also found a larger effect of longer sleep duration in Indian participants, which is consistent with results from a recent study in a similar population [57].

Although physical activity showed a positive relationship with T2D in this study, the association was not statistically significant. Self-report questionnaires can result in biased measurement of physical activity as they are highly dependent on participants’ memory and suffer from recall and reporting bias [58,59]. Objective measurements of physical activity, for example using a pedometer or accelerometer, provide more reliable results [60]. Indeed, poor correlation between self-reported physical activity and pedometer-assessed step count has been reported [61]. Taken together, these limitations may have caused over- or under-reporting of physical activity by participants in this study. The transition between two versions of physical activity questionnaires during the study may have also influenced results. However, differences are likely to be small, given that the difference between the original IPAQ and the modified IPAQ-M related only to language and cultural adaptation, based on the translation from English to Malay.

Another limitation is that this study did not incorporate dietary risk factors, although dietary factors have important effects on T2D risk. Dietary factors not assessed in this study but previously associated with T2D include polished rice and refined wheat, which are staple foods in Asia [62], sugar-sweetened beverages [63] and Western-style fast food [64], consumption of which has been rising in Asian countries. These may have a substantial influence on T2D risk in Malaysian populations, which we were not able to characterize using the available data. This was because dietary data, while collected, had not yet being processed at the time of this study. We also acknowledge the limitations inherent in using a nested case-control design for this study. While the design is efficient and cost-effective, it does not allow the estimation of incidence or relative risks, with associations expressed here as odds ratios. While traditional case-control studies can be subject to selection bias and exposure recall bias, the nested case-control design is less prone to these biases, due to cases and controls being selected from the same cohort [65].

The predictive accuracy of the seven-factor models across ancestral groups ranged from 0.76 to 0.85 (with 1 indicating perfect classification), indicating good to high discriminatory power. Although various predictive models have been constructed for T2D [18,66,67], there is limited data comparing different Asian populations, and even fewer studies assessing Malays. The AUC estimates were higher in Chinese than Malay and Indian. This substantially reflects the relatively larger effect of WHR in Chinese, as discussed above. This greater effect was also observed as a significant interaction between larger WHR and Chinese ancestry, suggesting that individuals of Chinese ancestry with higher WHR ratio may be an important subpopulation for targeted T2D interventions.

Although the statistical models performed well, a substantial proportion of T2D variation remained unexplained. Further, although this study showed that Chinese had higher odds of disease given the defined risk factors, the prevalence of T2D in Malaysia is highest in Indians. Ancestral differences in T2D prevalence may partly reflect ancestry-specific interactions between genetic and environmental factors [68] and gene-environment interaction studies may provide insights into T2D risk differences in the multiethnic Malaysian population. Another possible contributor is epigenetic modification [69], with studies reporting that DNA methylation is influenced by diet and exercise [70,71,72] and that methylation scores at T2D risk loci differ between Asians and Europeans [73].

## 5. Conclusions

Notwithstanding the above limitations, this study identified T2D risk factors shared across multiethnic Malaysian populations and identified differences in relative effects between ancestral groups. These results may help identify susceptible population subgroups for targeted intervention strategies, to help slow the increasing incidence of T2D in Malaysia. While age and family history are non-modifiable risk factors, interventions or public health campaigns could specifically target lifestyle change in older adults with a positive family history, and also those residing in rural areas. These findings also suggest the possible utility of ethnicity-specific anthropometric cut-points for quantifying diabetes risk.

## Figures and Tables

**Table 1:** Demographic and clinical characteristics of the Malaysian sample (n = 4077).

	Malay (n = 1323)		Chinese (n = 1344)		Indian (n = 1410)	
	T2D	Control	T2D	Control	T2D	Control
	n (%)	n (%)	n (%)	n (%)	n (%)	n (%)
**Gender**						
Male	285 (47.5) ***	252 (34.9)	343 (52.5) ***	153 (22.2)	401 (56.6) ***	221 (31.5
Female	315 (52.5)	471 (65.2)	311 (47.6)	537 (77.8)	307 (43.4)	481 (68.5)
**Age group, years**						
Less than 50	200 (33.3) ***	424 (58.6)	215 (32.9) ***	434 (62.9)	293 (41.4) ***	486 (69.2)
50–60	321 (53.5)	243 (33.6)	265 (40.5)	209 (30.3)	337 (47.6)	174 (24.8)
More than 60	79 (13.2)	56 (7.8)	174 (26.6)	47 (6.8)	78 (11.0)	42 (6.0)
**Location**						
Rural	164 (27.3) *	162 (22.4)	9 (1.4)	8 (1.2)	97 (13.7) ***	38 (5.4)
Urban	436 (72.7)	561 (77.6)	645 (98.6)	682 (98.8)	611 (86.3)	664 (94.6)
**Family History of Diabetes (FHD)**						
Negative	192 (32)	257 (35.6)	204 (33.6) **	291 (42.2)	148 (20.9) ***	199 (28.4)
Positive	408 (68)	466 (64.5)	403 (66.4)	399 (57.8)	560 (79.1)	501 (71.6)
**BMI category, kg/m^2^**						
Normal (<25)	153 (25.5) ***	266 (36.8)	260 (39.8) ***	510 (73.9)	241 (34.0)	271 (38.6)
Pre-obese (25–29.9)	268 (44.7)	313 (43.3)	267 (40.8)	152 (22.0)	292 (41.2)	275 (39.2)
Obese (>30)	179 (29.8)	144 (19.9)	127 (19.4)	28 (4.1)	175 (24.7)	156 (22.2)
**Waist-to-Hip Ratio (WHR)**						
Low risk (<0.95 M, <0.80 F)	223 (37.2) ***	392 (54.2)	232 (35.5) ***	407 (59.0)	207 (29.2) ***	315 (44.9)
Moderate risk (0.96–1 M, 0.81–0.85 F)	104 (17.3)	151 (20.9)	127 (19.4)	166 (24.1)	141 (19.9)	177 (25.2)
High risk (>1 M, >0.85 F)	273 (45.5)	180 (24.9)	295 (45.1)	117 (17.0)	360 (50.9)	210 (29.9)
**Physical activity** ^1^						
Active	44 (10.6)	52 (14.9)	17 (7.6)	30 (8.1)	64 (17.7)	77 (14.7)
Inactive	373 (89.5)	298 (85.1)	206 (92.4)	342 (91.9)	297 (82.3)	447 (85.3)
**Average sleep duration** ^1^						
Less than 6 h	83(19.9)	78(22.3)	26(11.7)	28(7.5)	52(14.4) ***	84(16.0)
6–7 h	159(38.1)	118(33.7)	38(17.0)	92(24.7)	95(26.3)	172(32.8)
7–8 h	93(22.3)	92 (26.3)	86(8.6)	145(39.0)	95(26.3)	136(26.0)
8–9 h	58(13.9)	39 (11.1)	52(23.3)	82(22.0)	73(20.2)	102(19.5)
9–10 h	17(4.1)	16 (4.6)	15(6.7)	17(4.6)	22(6.1)	24(4.6)
More than 10 h	7(1.7)	7(2.0)	6(2.7)	8(2.2)	24(6.7)	6(1.2)

^1^ n may not sum to sample size due to missing data: Malay = 767; Chinese = 595; Indian = 885. Denotes a statistically significant difference between cases and controls, at * *p* < 0.05; ** *p* < 0.01; *** *p* < 0.001.

**Table 2:** Odds ratios, 95% confidence intervals and *p*-values from complete case analysis (n = 2247).

	Complete-Case OR (95% CI)			
	Malays (767)	Chinese (595)	Indians (885)	Combined ^1^
Age 50–60 (Ref: <50)	2.48 (1.77, 3.48) *p* < 0.001	1.11 (0.70, 1.78) *p* = 0.652	2.62 (1.87, 3.68) *p* < 0.001	2.13 (1.73, 2.62) *p* < 0.001
Age > 60	1.95 (1.02, 3.72) *p* = 0.043	3.35 (1.79,6.24) *p* < 0.001	2.23 (1.24, 4.03) *p* = 0.008	2.71 (1.91, 3.85) *p* < 0.001
Gender: Female (Ref: Male)	1.15 (0.79,1.68) *p* = 0.469	1.57 (0.93, 2.65) *p* = 0.092	0.96 (0.66, 1.41) *p* = 0.851	1.13 (0.89, 1.42) *p* = 0.314
WHR	3.01 (2.30, 3.93) *p* < 0.001	6.28 (4.27, 9.25) *p* < 0.001	3.23 (2.46, 4.23) *p* < 0.001	3.63 (3.07, 4.28) *p* < 0.001
Physical inactivity (Ref: Physical activity)	1.21 (0.74, 2.00) *p* = 0.446	1.07 (0.49, 2.32) *p* = 0.874	0.94 (0.61, 1.45) *p* = 0.790	1.10 (0.82, 1.48) *p* = 0.538
Location: Rural (Ref: Urban)	1.67 (1.05, 2.67) *p* = 0.031	1.28 (0.29, 5.53) *p* = 0.744	2.6 (1.61, 4.22) *p* < 0.001	1.91 (1.39, 2.64) *p* < 0.001
Positive FHD (Ref: Negative FHD)	0.77 (0.52, 1.12) *p* = 0.175	1.90 (1.22, 2.96) *p* = 0.005	1.64 (1.13,2.38) *p* = 0.009	1.32 (1.06, 1.64) *p* = 0.013
Average sleep duration (Ref: 7–8 h)				
Less than 6 h	0.92 (0.58, 1.48) *p* = 0.740	0.89 (0.40, 1.96) *p* = 0.769	0.85 (0.51, 1.41) *p* = 0.534	0.83 (0.61, 1.13) *p* = 0.229
6–7 h	1.38 (0.91, 2.10) *p* = 0.128	0.59 (0.33, 1.05) *p* = 0.072	0.85 (0.56, 1.29) *p* = 0.449	0.94 (0.73, 1.21) *p* = 0.633
8–9 h	1.09 (0.62, 1.93) *p* = 0.771	0.80 (0.46, 1.39) *p* = 0.422	1.11 (0.69, 1.77) *p* = 0.673	0.98 (0.73, 1.31) *p* = 0.873
9–10 h	0.71 (0.31, 1.65); *p* = 0.432	0.87 (0.32, 2.36) *p* = 0.781	1.43 (0.69, 2.96) *p* = 0.333	0.98 (0.61, 1.58) *p* = 0.941
More than 10 h	0.61 (0.19, 1.98) *p* = 0.418	0.71 (0.19, 2.68) *p* = 0.610	7.10 (2.38, 21.17) *p* < 0.001	1.81 (0.93, 3.51) *p* = 0.079
**Pseudo R^2^ (%)**	0.16	0.29	0.22	0.21
**ROC (95% CI)**	0.76 (0.73, 0.80)	0.85 (0.81, 0.88)	0.81 (0.78, 0.83)	0.80 (0.78, 0.82)

^1^ Combinedmodel including ethnicity as a fixed effect. WHR = Waist-to-Hip Ratio, FHD = Family History of Diabetes.

**Table 3:** Odds ratios, 95% confidence intervals and *p*-values from multiply imputed data (n = 4077).

	Multiple Imputation OR (95% CI)			
	Malays (1323)	Chinese (1344)	Indians (1410)	Combined ^1^ (4077)
Age 50–60 (Ref: <50)	2.46 (2.30, 2.62) *p* < 0.001	1.19 (1.08, 1.30) *p* < 0.001	2.64 (2.47, 2.82) *p* < 0.001	2.14 (2.05, 2.23) *p* < 0.001
Age > 60 (Ref: <50)	1.97 (1.73, 2.23) *p* < 0.001	3.66 (3.25, 4.13) *p* < 0.001	2.08 (1.86, 2.33) *p* < 0.001	2.73 (2.55, 2.92) *p* < 0.001
Gender: Female (Ref: Male)	1.13 (1.05, 1.22) *p* = 0.001	1.51 (1.37, 1.67) *p* < 0.001	0.95 (0.88, 1.03) *p* = 0.204	1.11 (1.06, 1.16) *p* < 0.001
WHR	3.01 (2.85, 3.17) *p* < 0.001	6.01 (5.58, 6.47) *p* < 0.001	3.22 (3.05, 3.40) *p* < 0.001	3.60 (3.49, 3.72) *p* < 0.001
Physical inactivity (Ref: Physical activity)	1.19 (1.08, 1.32) *p* < 0.001	1.14 (0.98, 1.33) *p* = 0.093	1.01 (0.93, 1.10) *p* = 0.810	1.14 (1.07, 1.21) *p* < 0.001
Location: Rural (Ref: Urban)	1.71 (1.56, 1.88) *p* < 0.001	1.80 (1.35, 2.39) *p* < 0.001	2.51 (2.29, 2.76) *p* < 0.001	1.93 (1.81, 2.06) *p* < 0.001
Positive FHD (Ref: Negative FHD)	0.75 (0.70, 0.81) *p* < 0.001	1.88 (1.72, 2.05) *p* < 0.001	1.60 (1.48, 1.72) *p* < 0.001	1.30 (1.25, 1.81) *p* < 0.001
Average sleep duration (Ref: 7–8 h)				
Less than 6 h	0.89 (0.81, 0.98) *p* = 0.015	0.88 (0.76, 1.03) *p* = 0.106	0.88 (0.80, 0.97) *p* = 0.014	0.83 (0.78, 0.88) *p* < 0.001
6–7 h	1.39 (1.28, 1.51) *p* < 0.001	0.65 (0.58, 0.72) *p* < 0.001	0.85 (0.78, 0.93) *p* < 0.001	0.96 (0.91, 1.01) *p* = 0.106
8–9 h	1.11 (0.99, 1.24) *p* = 0.08	0.78 (0.70, 0.87) *p* < 0.001	1.10 (1.00, 1.21); *p* = 0.036	0.97 (0.91, 1.02) *p* = 0.236
9–10 h	0.72 (0.61, 0.85) *p* < 0.001	0.85 (0.70, 1.03) *p* = 0.10	1.42 (1.23, 1.64) *p* < 0.001	0.98 (0.89, 1.07) *p* = 0.653
More than 10 h	0.61 (0.49, 0.77) *p* < 0.001	0.76 (0.59, 0.97) *p* = 0.97	7.18 (5.79, 890) *p* < 0.001	1.83 (1.61, 2.08) *p* < 0.001
Pseudo R^2^ (%)	0.16	0.29	0.22	0.21
	0.76 (0.76, 0.77)	0.84 (0.84, 0.85)	0.80 (0.80, 0.81)	0.80 (0.79, 0.80)

^1^ Combined model including ethnicity as a fixed effect. WHR = Waist-to-Hip Ratio, FHD = Family.

**Table 4:** Area under the receiver operating characteristic curves (AUC) with 95% confidence interval (CI) for each ancestral group, and *p*-values from comparisons between groups.

AUC (95% CI)	Malay: 0.76 (0.73, 0.80)	Chinese: 0.84 (0.81, 0.88)	Indian: 0.80 (0.78, 0.83)	Global Test of All Ancestral Groups
**Malay**: 0.76 (0.73, 0.80)		*p* = 0.0004	*p* = 0.052	*p* = 0.0018
**Chinese**: 0.84 (0.81, 0.88)	*p* = 0.0004		*p* = 0.065	
**Indian**: 0.80 (0.78, 0.83)	*p* = 0.052	*p* = 0.065		

AUC estimates derived from logistic models including gender, age, waist-to-hip ratio and physical activity.

**Table 5:** Interaction of risk factor effects with ancestral group.

Interaction	OR (95% CI) *p*-Value
**Age group by Ancestral group** (Ref: Aged < 50, Malay)	
Aged 50–60 years by Chinese	0.52 (0.32, 0.85) *p* = 0.009
Aged 50–60 years by Indian	1.06 (0.69, 1.62) *p* = 0.804
Aged more than 60 years by Chinese	2.06 (0.93, 4.57) *p* = 0.075
Aged more than 60 years by Indian	0.95 (0.44, 2.06) *p* = 0.891
**Gender by Ancestral group** (Ref: Male, Malay)	
Female by Chinese	0.5 (0.32, 0.8) *p* = 0.003
Female by Indian	0.6 (0.4, 0.89) *p* = 0.011
**Waist-to-Hip Ratio by Ancestral group** (Ref: Low risk (<0.95 M, <0.80 F), Malay)	
Moderate risk (0.96–1 M, 0.81–0.85 F) by Chinese	1.15 (0.64, 2.07) *p* = 0.630
Moderate risk (0.96–1 M, 0.81–0.85 F) by Indian	1.11 (0.66, 1.88) *p* = 0.694
High risk (>1 M, >0.85 F) by Chinese	2.10 (1.23, 3.57) *p* = 0.006
High risk (>1 M, >0.85 F) by Indian	1.24 (0.78, 1.97) *p* = 0.362
**Physical Activity by Ancestral group** (Ref: Active, Malay)	
Inactive by Chinese	0.72 (0.34, 1.53) *p* = 0.390
Inactive by Indian	0.54 (0.31, 0.95) *p* = 0.032
**Locality by Ancestral groups** (Ref: Urban, Malay)	
Rural by Chinese	0.93 (0.3, 2.94) *p* = 0.905
Rural by Indian	1.51 (0.84, 2.73) *p* = 0.167
**Family History of T2D by Ancestral group** (Ref: Negative FHD, Malay)	
Positive FHD by Chinese	2.96 (1.82, 4.81) *p* < 0.001
Positive FHD by Indian	2.43 (1.53, 3.85) *p* < 0.001
**Sleep duration categories by Ancestral group** (Ref: Sleep duration 7–8 h, Malay)	
Sleep duration less than 6 h by Chinese	1.49 (0.72, 3.09) *p* = 0.287
Sleep duration less than 6 h by Indian	0.84 (0.46, 1.54) *p* = 0.578
Sleep duration 6–7 h by Chinese	0.52 (0.29, 0.95) *p* = 0.032
Sleep duration 6–7 h by Indian	0.59 (0.35, 1.00) *p* = 0.049
Sleep duration 8–9 h by Chinese	0.73 (0.37, 1.41) *p* = 0.345
Sleep duration 8–9 h by Indian	0.7 (0.37, 1.32) *p* = 0.266
Sleep duration 9–10 h by Chinese	1.42 (0.50, 4.04) *p* = 0.517
Sleep duration 9–10 h by Indian	1.25 (0.47, 3.31) *p* = 0.656
Sleep duration more than 10 h by Chinese	1.28 (0.27, 5.96) *p* = 0.755
Sleep duration more than 10 h by Indian	5.79 (1.38, 24.23) *p* = 0.016

WHR = Waist-to-Hip Ratio, FHD = Family History of Diabetes.

## Supplementary Materials

The following are available online at http://www.mdpi.com/1660-4601/15/12/2813/s1, Table S1: Demographic and clinical characteristics of the sample used in complete case analyses (n = 2247). Table S2: Odds ratio and 95% confidence interval for secondary analysis (age, categorical waist-to-hip ratio and physical activity) based on complete-case data. Table S3: Odds ratio and 95% confidence interval for secondary analysis (age, categorical waist-to-hip ratio and physical activity) based on multiply imputed data. Table S4: Positive association between locality and obesity.
