# Supplementary material for: Differing Contributions of Classical Risk Factors to Type 2 Diabetes in Multi-Ethnic Malaysian Populations
Source: Int J Environ Res Public Health. 2018 Dec 10;15(12):2813. doi: 10.3390/ijerph15122813 (PMC6313591; doi:10.3390/ijerph15122813)
Supplement: Supplementary file 1 [file ijerph-15-02813-s001.pdf]

Data Supplement

List of moderate physical activities

At workplace:

1. Lifting moderate loads (5kg)
2. Lifting boxes
3. Mending
4. Hoeing weeds
5. Mowing
6. Gardening
7. Going up and downs stairs

At home:

1. Hoeing weeds
2. Gardening
3. Cleaning outside house
4. Raking
5. Mowing
6. Lifting moderate loads

Recreational:

1. Aerobic
2. Playing sport such as badminton, basketball, volleyball, bowling, table-tennis, golfing
3. Cycling
4. Dancing
5. Swimming
6. Brisk- walking
7. Yoga
8. Walking on treadmill
9. Qiqong
10. Tai-chi
11. Yoga
12. Heavy lifting gym

List of vigorous physical activities

At workplace:

1. Loading things into trucks
2. Lifting heavy things 7-18kg
3. Lifting heavy things upstairs
4. Using heavy tools (drilling, digging)
5. Digging trench

At home:

1. Moving and lifting furniture
2. Lifting things upstairs
3. Lifting heavy boxes
4. Hoeing weeds
5. Going ups and downs stairs

Recreational:

1. Aerobic
2. Cycling
3. Swimming
4. Playing sports such as badminton, football, volleyball, hockey, tennis, rugby
5. Jogging
6. Hiking
7. Martial arts, self-defence such as karate, judo, taekwondo

Table S1: Demographic and clinical characteristics of participants included in complete case analyses (n=2247)

	Malay (N=767)			Chinese (N=595)			Indian (N=885)		
	T2D N (%)	Control N (%)	Total N (%)	T2D N (%)	Control N (%)	Total N (%)	T2D N (%)	Control N (%)	Total N (%)
**Gender**									
Male	205(49.16)**	130(37.14)	335(43.68)	116(52.02)***	93(25)	209(35.13)	210(58.17)***	176(33.59)	386(43.62)
Female	212(50.84)	220(62.86)	432(56.32)	107(47.98)	279(75)	386(64.87)	151(41.83)	348(66.41)	499(56.38)
**Age group, years**									
Less than 50	159(38.13)***	229(65.43)	388(50.59)	84(37.67)***	220(59.14)	304(51.09)	152(42.11)***	366(69.85)	518(58.53)
50-60	217(52.04)	103(29.43)	320(41.72)	77(34.53)	128(34.41)	205(34.45)	169(46.81)	127(24.24)	296(33.45)
More than 60	41(9.83)	18(5.14)	59(7.69)	62(27.8)	24(6.45)	86(14.45)	40(11.08)	31(5.92)	71(8.02)
**Location**									
Rural	91(21.82)***	36(10.29)	127(16.56)	8(3.59)	6(1.61)	14(2.35)	79(21.88)***	37(7.06)	116(13.11)
Urban	326(78.18)	314(89.71)	640(83.44)	215(96.41)	366(98.39)	581(97.65)	282(78.12)	487(92.94)	769(86.89)
**Family history**									
Yes	121(29.02)**	71(20.29)	192(25.03)	69(33.01)**	177(47.58)	246(42.34)	76(21.05)**	150(28.74)	226(25.59)
No	296(70.98)	279(79.71)	575(74.97)	140(66.99)	195(52.42)	335(57.66)	285(78.95)	372(71.26)	657(74.41)
**BMI category, kg/m2**									
Normal (<25)	115(27.58)*	123(35.14)	238(31.03)	70(31.39)***	264(70.97)	334(56.13)	117(32.41)	201(38.36)	318(35.93)
Pre-obese (25-29.9)	180(43.17)	155(44.29)	335(43.68)	110(49.33)	91(24.46)	201(33.78)	150(41.55)	205(39.12)	355(40.11)
Obese (>30)	122(29.26)	72(20.57)	194(25.29)	43(19.28)	17(4.57)	60(10.08)	94(26.04)	118(22.52)	212(23.95)

**Waist-to-Hip Ratio**									
Low risk (<0.95 M, <0.80 F)	163(39.09)***	187(53.43)	350(45.63)	76(34.08)***	220(59.14)	296(49.75)	99(27.42)***	238(45.42)	337(38.08)
Moderate risk (0.96-1 M, 0.81-0.85 F)	79(18.94)	82(23.43)	161(20.99)	41(18.39)	93(25)	134(22.52)	69(19.11)	135(25.76)	204(23.05)
High risk (>1 M, >0.85 F)	175(41.97)	81(23.14)	256(33.38)	106(47.53)	59(15.86)	165(27.73)	193(53.46)	151(28.82)	344(38.87)
**Physical activity**[a]									
Active	44(10.55)	52(14.86)	96(12.52)	17(7.62)	30(8.06)	47(7.9)	64(17.73)	77(14.69)	141(15.93)
Inactive	373(89.45)	298(85.14)	671(87.48)	206(92.38)	342(91.94)	548(92.1)	297(82.27)	447(85.31)	744(84.07)
**Average sleep duration**[a]									
Less than 6 hours	93(22.3)	92(26.29)	185(24.12)	86(38.57)	145(38.98)	231(38.82)	95(26.32)**	136(25.95)	231(26.1)
6-7 hours	83(19.9)	78(22.29)	161(20.99)	26(11.66)	28(7.53)	54(9.08)	52(14.4)	84(16.03)	136(15.37)
7-8 hours	159(38.13)	118(33.71)	277(36.11)	38(17.04)	92(24.73)	130(21.85)	95(26.32)	172(32.82)	267(30.17)
8-9 hours	58(13.91)	39(11.14)	97(12.65)	52(23.32)	82(22.04)	134(22.52)	73(20.22)	102(19.47)	175(19.77)
9-10 hours	17(4.08)	16(4.57)	33(4.3)	15(6.73)	17(4.57)	32(5.38)	22(6.09)	24(4.58)	46(5.2)
More than 10 hours	7(1.68)	7(2)	14(1.83)	6(2.69)	8(2.15)	14(2.35)	24(6.65)	6(1.15)	30(3.39)

Denotes statistically significant at *P<0.05; ** P<0.01; ***P<0.001

Table S2: Odds ratio and 95% confidence interval for secondary analysis (using categorical waist-to-hip ratio) based on complete-case data

	OR (95% CI)			
	**Malays (767)**	**Chinese (595)**	**Indians (885)**	**Combined[1] (2247)**
**Age 50-60 (Ref: <50)**	2.73 (1.98 ,3.76) P<0.001	1.33 (0.87, 2.03) P=0.19	3.18 (2.31, 4.37) P<0.001	2.48 (2.04, 3.01) P<0.001
**Age>60 (Ref:<50)**	2.72 (1.47, 5.03) P=0.001	5.14 (2.87, 9.21) P<0.001	2.95 (1.68, 5.18) P<0.001	3.80 (2.73, 5.30) P<0.001
**WHR: Moderate risk (Ref: Low risk)**	1.09 (0.74, 1.62) P=0.659	1.25 (0.76, 2.04) P=0.38	1.13 (0.76, 1.69) P=0.54	1.11 (0.88, 1.41) P=0.38
**WHR: High risk (Ref: Low risk)**	2.17 (1.51, 3.12) P<0.001	4.62 (2.95,7.23) P<0.001	2.43 (1.73, 3.43) P<0.001	2.66 (2.15, 3.28) P<0.001
**Physical inactivity (Ref: Physical activity)**	1.04 (0.66, 1.65) P=0.86	0.89 (0.44, 1.78) P=0.74	0.70 (0.47, 1.04) P=0.08	0.86 (0.65, 1.13) P=0.27
**Location: Urban (Ref:Rural)**	1.81 (1.15, 2.83) P=0.01	2.29 (0.67, 7.83) P=0.19	3.33 (2.12, 5.22) P<0.001	2.35 (1.73, 3.19) P<0.001
**Family history of DM: Yes (Ref: No)**	0.69 (0.48, 0.99) P=0.04	1.83 (1.23, 2.72) P=0.003	1.75 (1.23, 2.48) P=0.002	1.29 (1.05, 1.58) P=0.014
**Average sleep duration (Ref: 7-8 hours)**				
Less than 6 hours	1.00 (0.64, 1.57) P=0.99	1.29 (0.66, 2.56) P=0.46	0.89 (0.55, 1.43) P=0.62	0.94 (0.70, 1.25) P=0.65
6-7 hours	1.39 (0.93, 2.07) P=0.11	0.71 (0.42, 1.21) P=0.20	0.79 (0.53 , 1.18) P=0.25	0.95 (0.75, 1.21) P=0.68
8-9 hours	1.28 (0.75, 2.18) P=0.37	1.10 (0.67, 1.81) P=0.71	0.98 (0.63, 1.52) P=0.94	1.06 (0.80, 1.39) P=0.69
9-10 hours	0.92 (0.42, 2.04) P=0.84	1.31 (0.55, 3.16) P=0.54	1.21 (0.60, 2.42) P=0.59	1.10 (0.70, 1.71) P=0.68
More than 10 hours	0.77 (0.24, 2.41) P=0.65	1.09 (0.31, 3.78) P=0.89	4.60 (1.67, 12.66) P=0.003	1.84 (0.99, 3.42) P=0.054
**Pseudo R^2 (%)**	0.09	0.16	0.14	0.12
**ROC (95% CI)**	0.70 (0.66, 0.73)	0.76 (0.72, 0.80)	0.74 (0.71, 0.78)	0.73 (0.71, 0.75)

[1] Combined model adjusted for ethnicity.

Table S3: Odds ratio and 95% confidence interval for secondary analysis (using categorical waist-to-hip ratio) based on multiply imputed data

	OR (95% CI)			
	**Malays (1323)**	**Chinese (1344)**	**Indians (1410)**	**Combined (4077)**
**Age 50-60 (Ref: <50)**	2.73 (2.56, 2.90) P<0.001	1.40 (1.29, 1.52) P<0.001	3.16(2.97, 3.37) P<0.001	2.48 (2.39, 2.58) P<0.001
**Age>60 (Ref:<50)**	2.75 (2.44, 3.10) P<0.001	5.67 (5.06, 6.34) P<0.001	2.74 (2.46, 3.05) P<0.001	3.82 (3.58, 4.07) P<0.001
**WHR: Moderate risk (Ref: Low risk)**	1.08 (1.00, 1.17) P=0.051	1.14 (1.03, 1.25) P=0.009	1.16 (1.07, 1.26) P<0.001	1.10 (1.05, 1.15) P<0.001
**WHR: High risk (Ref: Low risk)**	2.12 (1.97, 2.27) P<0.001	4.32 (3.96, 4.71) P<0.001	2.47 (2.30, 2.64) P<0.001	2.61 (2.50, 2.72) P<0.001
**Physical inactivity (Ref: Physical activity)**	1.04 (0.95, 1.13) P=0.447	0.95 (0.83, 1.09) P=0.438	0.73 (0.67, 0.79) P<0.001	0.88 (0.84, 0.93) P<0.001
**Location: Urban (Ref:Rural)**	0.54 (0.50, 0.59) P<0.001	0.30 (0.24, 0.39) P<0.001	0.32 (0.29, 0.34) P<0.001	0.42 (0.40, 0.45) P<0.001
**Family history of DM: Yes (Ref: No)**	0.68 (0.64, 0.73) P<0.001	1.81 (1.68, 1.95) P<0.001	1.73 (1.61, 1.86) P<0.001	1.29 (1.24, 1.34) P<0.001
**Average sleep duration (Ref: 7-8 hours)**				
Less than 6 hours	0.99 (0.91, 1.08) P=0.86	1.25 (1.10, 1.43) P=0.001	0.93 (0.85, 1.02) P=0.124	0.95 (0.90, 1.00) P=0.064
6-7 hours	1.39 (1.29, 1.51) P<0.001	0.77 (0.69, 0.85) P<0.001	0.79 (0.73, 0.85) P<0.001	0.96 (0.92, 1.01) P=0.137
8-9 hours	1.29 (1.17, 1.44) P<0.001	1.07 (0.97, 1.18) P=0.157	0.97 (0.89, 1.06) P=0.543	1.04 (0.99, 1.10) P=0.121
9-10 hours	0.93 (0.80, 1.09) P=0.378	1.15 (0.97, 1.37) P=0.108	1.20 (1.05, 1.38) P=0.008	1.07 (0.98, 1.16) P=0.143
More than 10 hours	0.77 (0.62, 0.97) P=0.025	1.18 (0.93, 1.49) P=0.168	4.64 (3.80, 5.66) P<0.001	1.87 (1.66, 2.11) P<0.001
**Pseudo R^2 (%)**	0.09	0.16	0.14	0.12
**ROC (95% CI)**	0.70 (0.69, 0.70)	0.76 (0.76, 0.77)	0.74 (0.73, 0.75)	0.73 (0.72, 0.73)

Table S4: Positive association between locality and obesity

**Obesity**	**Rural**	**Urban**	**P**
Normal	79 (30.74)	811 (40.75)	0.007
Pre-obese	113 (43.97)	778 (39.10)	
Obese	65 (25.29)	401 (20.15)	
